# Supplementary material for: Hot spot prediction in protein-protein interactions by an ensemble system
Source: BMC Syst Biol. 2018 Dec 31;12(Suppl 9):132. doi: 10.1186/s12918-018-0665-8 (PMC6311905; doi:10.1186/s12918-018-0665-8)
Supplement: Supplementary file 2 — All relevant MATLAB code. A simple Matlab implement of our predictor. (ZIP 19,742 kb) [file 12918_2018_665_MOESM2_ESM.zip › Additional file 2/Read ASA/ASA website/SKEMPI/1FC2C.htm]

 NetSurfP - Protein Surface Accessibility and Secondary Structure Predictions

  

|  |  |  |
| --- | --- | --- |
|  |  | NetSurfP - Protein Surface Accessibility and Secondary Structure Predictions  Technical University of Denmark |

  


---

```
# For publication of results, please cite:
# A generic method for assignment of reliability scores applied to solvent accessibility predictions.
# Bent Petersen, Thomas Nordahl Petersen, Pernille Andersen, Morten Nielsen and Claus Lundegaard
# BMC Structural Biology 2009, 9:51 doi:10.1186/1472-6807-9-51
#
# Column 1: Class assignment - B for buried or E for Exposed - Threshold: 25% exposure, but not based on RSA
# Column 2: Amino acid
# Column 3: Sequence name
# Column 4: Amino acid number
# Column 5: Relative Surface Accessibility - RSA
# Column 6: Absolute Surface Accessibility
# Column 7: Z-fit score for RSA prediction
# Column 8: Probability for Alpha-Helix
# Column 9: Probability for Beta-strand
# Column 10: Probability for Coil
E F  1FC2C                  1    0.691 138.644  -2.136   0.003   0.003   0.994
E N  1FC2C                  2    0.649  95.058   0.117   0.115   0.016   0.868
E K  1FC2C                  3    0.619 127.267   0.322   0.717   0.014   0.269
E E  1FC2C                  4    0.656 114.656  -0.080   0.717   0.014   0.269
E Q  1FC2C                  5    0.406  72.422  -0.488   0.660   0.049   0.291
E Q  1FC2C                  6    0.438  78.227  -0.135   0.561   0.047   0.393
E N  1FC2C                  7    0.519  75.923  -0.868   0.502   0.102   0.396
B A  1FC2C                  8    0.236  25.985  -0.400   0.278   0.093   0.628
B F  1FC2C                  9    0.207  41.605  -1.773   0.199   0.152   0.649
B Y  1FC2C                 10    0.187  39.898  -0.236   0.135   0.317   0.548
E E  1FC2C                 11    0.366  63.923  -0.127   0.159   0.516   0.325
B I  1FC2C                 12    0.076  14.079   0.044   0.172   0.690   0.138
B L  1FC2C                 13    0.095  17.358   0.192   0.087   0.683   0.230
E H  1FC2C                 14    0.307  55.861  -0.656   0.022   0.552   0.426
B L  1FC2C                 15    0.093  16.973   0.803   0.019   0.141   0.840
E P  1FC2C                 16    0.339  48.062  -0.674   0.005   0.045   0.951
E N  1FC2C                 17    0.580  84.956  -1.030   0.005   0.045   0.951
B L  1FC2C                 18    0.148  27.135   0.090   0.018   0.019   0.964
E N  1FC2C                 19    0.469  68.632   0.563   0.016   0.005   0.979
E E  1FC2C                 20    0.611 106.654  -0.377   0.858   0.002   0.139
E E  1FC2C                 21    0.718 125.522  -0.905   0.782   0.003   0.216
E Q  1FC2C                 22    0.374  66.850  -0.303   0.694   0.003   0.303
B R  1FC2C                 23    0.234  53.655  -0.151   0.717   0.014   0.269
B N  1FC2C                 24    0.306  44.784  -0.905   0.660   0.049   0.291
B G  1FC2C                 25    0.269  21.170  -0.576   0.605   0.105   0.290
B F  1FC2C                 26    0.103  20.672  -0.013   0.701   0.107   0.192
B I  1FC2C                 27    0.091  16.835   0.234   0.649   0.163   0.188
E Q  1FC2C                 28    0.456  81.370   0.587   0.649   0.163   0.188
E S  1FC2C                 29    0.339  39.789  -0.075   0.605   0.105   0.290
B L  1FC2C                 30    0.114  20.800   0.216   0.386   0.097   0.517
E K  1FC2C                 31    0.511 105.092   0.111   0.191   0.086   0.723
E D  1FC2C                 32    0.569  81.950  -0.600   0.053   0.043   0.903
E D  1FC2C                 33    0.437  62.943  -0.238   0.018   0.019   0.964
E P  1FC2C                 34    0.432  61.272  -1.100   0.176   0.004   0.820
E S  1FC2C                 35    0.583  68.304  -1.014   0.321   0.003   0.675
E Q  1FC2C                 36    0.503  89.800  -0.078   0.502   0.002   0.495
E S  1FC2C                 37    0.366  42.907  -1.264   0.522   0.016   0.462
B A  1FC2C                 38    0.297  32.696  -1.002   0.522   0.016   0.462
E N  1FC2C                 39    0.510  74.620  -0.487   0.561   0.047   0.393
B L  1FC2C                 40    0.249  45.537   0.178   0.455   0.046   0.498
E L  1FC2C                 41    0.267  48.869  -0.398   0.455   0.046   0.498
E A  1FC2C                 42    0.497  54.813  -1.070   0.386   0.097   0.517
E E  1FC2C                 43    0.680 118.813   0.007   0.268   0.043   0.689
E A  1FC2C                 44    0.808  88.998  -1.041   0.003   0.003   0.994

Explain the output.  Go back.
```

---
